# Terawatt-scale optical half-cycle attosecond pulses

**DOI:** 10.1038/s41598-018-21052-2

**Published:** 2018-02-08

**Authors:** Jiancai Xu, Baifei Shen, Xiaomei Zhang, Yin Shi, Liangliang Ji, Lingang Zhang, Tongjun Xu, Wenpeng Wang, Xueyan Zhao, Zhizhan Xu

**Affiliations:** 1 State Key Laboratory of High Field Laser Physics, Shanghai Institute of Optics and Fine Mechanics, Chinese Academy of Sciences, P. O. Box 800-211, Shanghai, 201800 China; 2 Department of Physics, Shanghai Normal University, Shanghai, 200234 China; 3 Collaborative Innovation Center of IFSA (CICIFSA), Shanghai Jiao Tong University, Shanghai, 200240 China

## Abstract

Extreme-ultravoilet (XUV) attosecond pulses with durations of a few tens of attosecond have been successfully applied for exploring ultrafast electron dynamics at the atomic scale. But their weak intensities limit the further application in demonstrating nonlinear responses of inner-shell electrons. Optical attosecond pulses will provide sufficient photon flux to initiate strong-field processes. Here we proposed a novel method to generate an ultra-intense isolated optical attosecond pulse through relativistic multi-cycle laser pulse interacting with a designed gas-foil target. The underdense gas target sharpens the multi-cycle laser pulse, producing a dense layer of relativistic electrons with a thickness of a few hundred nanometers. When the dense electron layer passes through an oblique foil, it emits single ultra-intense half-cycle attosecond pulse in the visible and ultraviolet spectral range. The emitted pulse has a peak intensity exceeding 10^18^ W/cm^2^ and full-width-half-maximum duration of 200 as. The peak power of this attosecond light source reaches 2 terawatt. The proposed method relaxes the single-cycle requirement on the driving pulse for isolated attosecond pulse generation and significantly boosts the peak power, thus it may open up the route to new experiments tracking the nonlinear response of inner-shell electrons as well as nonlinear attosecond phenomena investigation.

## Introduction

Attosecond pulses can reveal electron dynamics at the atomic scale, and the attosecond techniques has undergone remarkable development in the past decade^1–3^. They offer an ideal tool with unprecedented time resolution to observe and control the microscopic motion of electrons in atoms, molecules, and nanostructures, and thus can assist the understanding of the fundamental processes in matter^4,5^. In general, ultrafast ‘pump-probe’ experiments take advantage of the extremely short temporal resolution of the attosecond pulse. If the photon flux of the attosecond light source, especially the isolated attosecond pulse, significantly increases, it will greatly extend its application for nonlinear ultrafast process investigations^6–8^.

Significant amounts of studies have been focused on increasing the peak power of attosecond pulses. In the traditional high-order harmonic generation from a gas target^9–11^, a loose-focusing geometry has been used to enhance the conversion efficiency, leading to microjoule-energy trains of extreme-ultravoilet (XUV) attosecond pulses. In the laser-solid interaction scenario, multi-hundred terawatts (TWs) laser systems are available to generate relativistic multi-cycle femtosecond pulses, which drive bright XUV/X-ray attosecond pulses in the sub-millijoule energy scale from the solid interface^12–15^.

**Figure 1:**
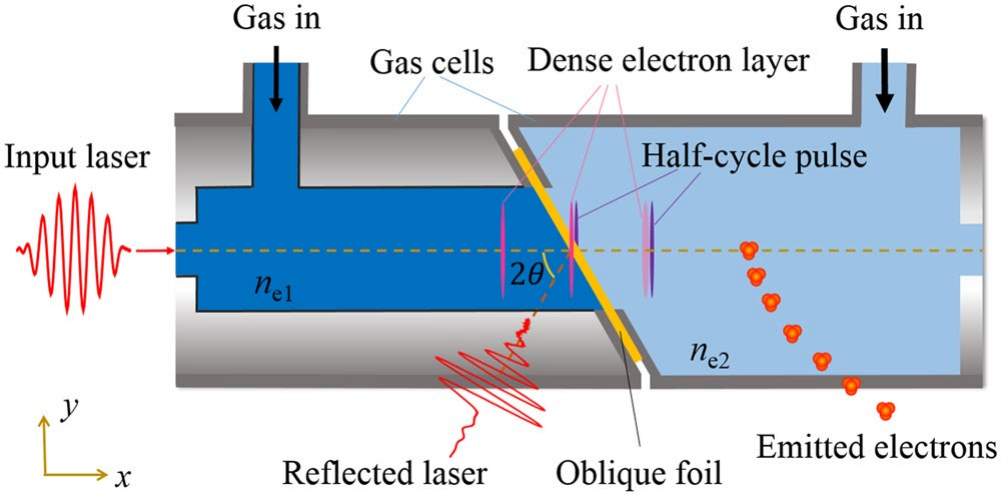
Physical scheme of half-cycle attosecond pulse emission and detection. A relativistic multi-cycle laser pulse shots into a gas-foil target (${n}_{{\rm{e}}1}$ gas cell and an oblique foil) and produces isolated ultra-intense half-cycle attosecond pulse. The generated attosecond pulse will be detected based on the second gas cell (${n}_{{\rm{e}}2}$ region) through the measurement of emitted electrons.

In this paper, we propose a novel scheme to produce isolated attosecond pulses with a peak power of 2.1 TW in the visible and ultraviolet spectral range by a specially designed target geometry. The produced optical attosecond pulse has a half-cycle structure in time domain with a duration of 200 as and reaches a peak intensity of 10^18^ W/cm^2^, which is several orders of magnitude more intense than that from a light-field synthesizer (~10^13^ W/cm^2^, ref.^16^). The intense optical attosecond pulse is emitted from a dense relativistic electron layer with a thickness of a few hundred nanometers as shown in Fig. 1. A multi-cycle laser pulse propagates along the *x*-axis in the underdense gas target (${n}_{{\rm{e}}1}$ region), realized based on a specially-designed gas cell^17^. Due to the laser shaping effect in the underdense plasma^18^, the initial multi-cycle laser pulse transforms to a step-like pulse and drives an ultra-thin dense layer of relativistic electrons sitting in the laser front. The key set-up in our proposal is a flat foil obliquely placed as referred to the laser propagation axis. When the dense relativistic electron layer passes through that oblique foil, an intense transverse current is triggered, emitting an optical half-cycle pulse with a duration of a few hundred attosecond at the rear side of the oblique foil^15,19^. Moreover, a simple way to detect the generated ultra-intense half-cycle pulse is present with the second low-density gas cell (${n}_{{\rm{e}}2}$ region). The high-intensity unipolar half-cycle pulse maintains its temporal structure in low-density gas and accelerates background electrons to relativistic energies with asymmetric angular distribution. The half-cycle pulse can be verified through the energy and emitted angle measurement of accelerated electrons.

The fundamental scheme of the ultra-intense half-cycle attosecond pulse generation was studied using the two-dimensional particle-in-cell simulation code VORPAL^20^. Simulation results indicate that the optical attosecond pulses were generated with different laser-target parameters. Here we take one laser-target example to describe the generation of ultra-intense optical half-cycle pulse, and the simulation parameters are present here. A moving simulation window with a size of 50 μm × 60 μm in the *x-y* plane is divided into 15000 × 1200 cells, where the time step of $3.3\,\mathrm{nm}/c$ is fine enough to resolve the attosecond features of the radiated pulse. The employed underdense plasma has a homogenous electron distribution with a density of ${n}_{{\rm{e}}1}=1\times {10}^{20}\,{{\rm{cm}}}^{-3}$. A *p*-polarized laser pulse along the *y*-axis propagates from the left boundary of the simulation box along *x*-axis. The typical employed incident laser pulse has a temporal profile of $a={a}_{0}\,\sin ({\rm{\pi }}t/{\tau }_{0})$, where the pulse duration is ${\tau }_{0}=8\,{\rm{fs}}$ and the dimensionless laser amplitude is ${a}_{0}=e{E}_{0}/{m}_{{\rm{e}}}{\omega }_{0}c=10$. Here ${E}_{0}$ is the peak incident laser field, ${\omega }_{0}$ is the central laser frequency,$\,e$ and ${m}_{{\rm{e}}}$ are the electron charge and mass, respectively, and $c$ is the light speed in vacuum. The laser shaping process in the underdense plasma depends on the initial laser pulse duration, where it takes more time for the case of long pulse duration^18^. To minimize the computation time in the high-resolution runs, we chose a relatively small initial laser pulse duration of 8 fs. The laser pulse has a focal spot size of 15 μm, around 5 times larger than the plasma wavelength in the ${n}_{{\rm{e}}1}$ plasma. The oblique flat foil target is located at *x* = 45 μm with a thickness of *d* = 2 μm and bias angle of $\theta =45^\circ$. It is fully ionized with an electron density of ${n}_{{\rm{e}},{\rm{f}}}=50\,{n}_{{\rm{c}}}$, where ${n}_{{\rm{c}}}=1.74\times {10}^{21}\,{{\rm{cm}}}^{-3}$ is the critical density for the central wavelength of the incident laser ${\lambda }_{0}=800\,\mathrm{nm}$.

**Figure 2:**
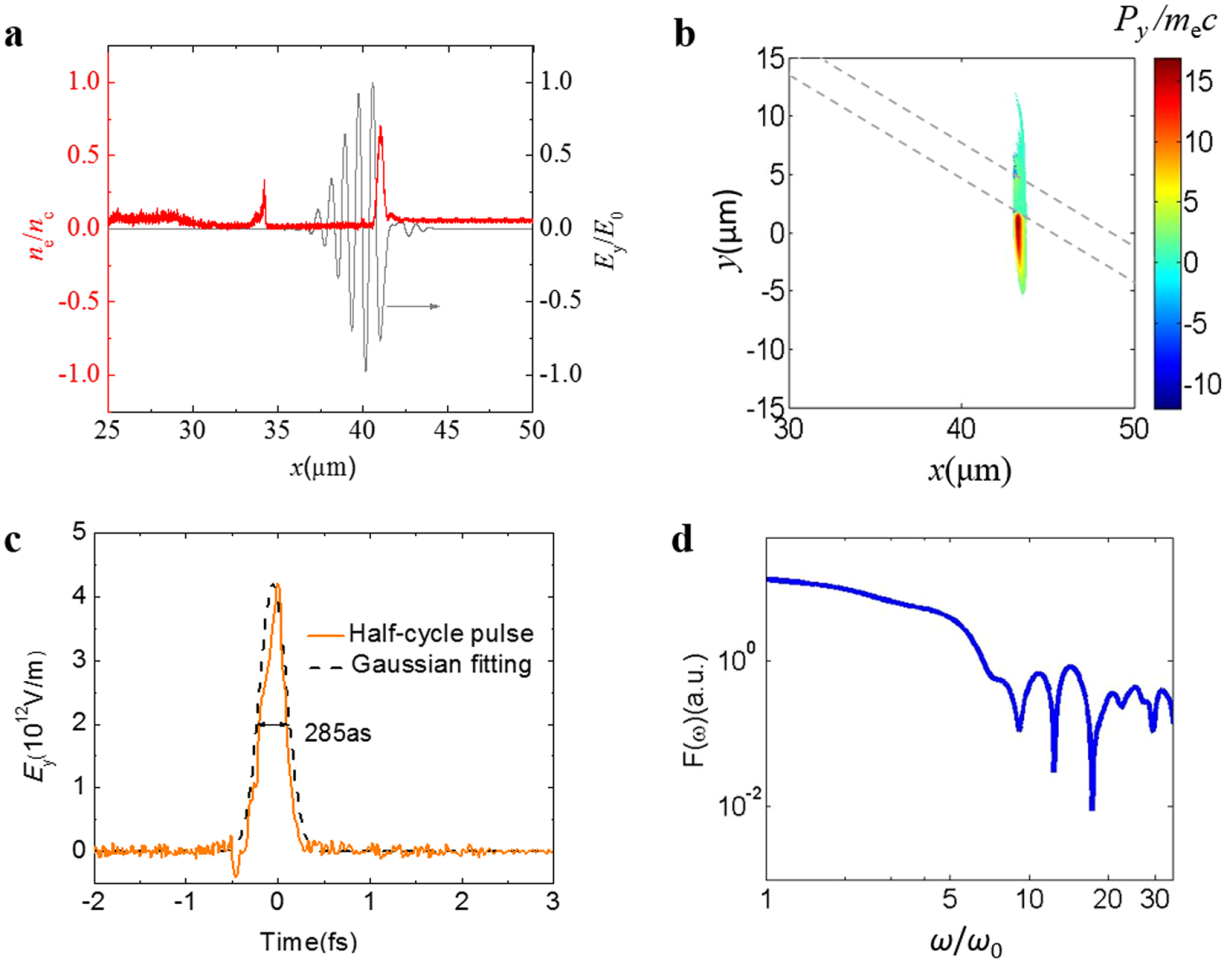
Dense layer of relativistic electrons and the generated ultra-intense optical attosecond pulse. (**a**) lineout of the electric field of the laser pulse (normalized to ${E}_{0}$) and the electron density (normalized to ${n}_{{\rm{c}}}$) at the transversal position of $y=0$ before the laser pulse reaches the oblique foil at $t=50\,{\rm{\mu }}{\rm{m}}/{\rm{c}}$. (**b**) Spatial distribution of transverse momentum ${P}_{y}\,$of the relativistic electron layer when passing through the oblique foil (foil is marked by dashed lines). (**c**) The electric field ${E}_{y}$ of the emitted optical attosecond pulse as well as the Gaussian fitting profile. (**d**) Spectrum of the half-cycle attosecond pulse at *t* = 100 μm/c, where ${\omega }_{0}$ is the laser frequency at the central wavelength of 800 nm.

The underdense plasma $\,{n}_{{\rm{e}}1}=1\times {10}^{20}\,{{\rm{cm}}}^{-3}$ shapes the initial multi-cycle laser pulse to a steep laser pulse and produces a dense layer of relativistic electrons, which is essential for isolated ultra-intense attosecond pulse generation. When the femtosecond laser pulse propagates in the $\,{n}_{{\rm{e}}1}$ plasma, the laser front is continuously etched backward and is steepened gradually, and this effect is referred to as the laser shaping effect^18^ or self-etching effect^21^. Therefore, after propagating for a certain distance in the underdense plasma, the laser shaping effect will cut off the laser front, resulting in a step-like pulse. For our simulation case, the initial pulse duration of the employed laser pulse is 8 fs, the electric field of the resulted step-like pulse is plotted in Fig. 2a. Meanwhile, the laser pulse maintains its high intensity (${E}_{y}/{E}_{0} \sim 1)$ because the self-focusing effect compensates the laser energy depletion, here ${E}_{0}$ is the initial peak laser amplitude. Such a step-like intense pulse poses a longitudinal ponderomotive force so strong in the first half laser cycle that it pushes up a huge number of background electrons and generates a density spike in that position, i.e. a highly-dense electron layer is formed^21^. Figure 2a displays the density plot of the electron layer along the longitudinal direction and it shows that the density spike has a peak value of 0.7 ${n}_{{\rm{c}}}$ with a narrow full-width-half-maximum (FWHM) thickness of 320 nm. Moreover, this electron layer has good transverse uniformity over a length of more than 10 μm because of the large laser focal spot size. The electron layer carries a total charge of more than 3 nC assuming that the electron layer is symmetric in the transverse *y-z* plane. As the initial laser peak intensity and its focal spot size get matched in the underdense plasma, the formed dense electron layer keeps its narrow thickness and high-density peak for a propagation distance of a few tens of micrometers since self-focusing effect and energy depletion will maintain the laser peak intensity and transverse size for a relatively long distance^22^. The electron layer formation depends on the laser pulse evolution in the ${n}_{{\rm{e}}1}$ gas target and requires high-intensity of initial laser pulse.

When the dense electron layer forms, it is positioned in the laser front and propagates along with the laser pulse. These electrons inside the layer are directly accelerated by the driving laser pulse, where the electron motion follows the single-electron picture in the local laser electric field^23^. The longitudinal momentum is ${P}_{x}/{m}_{{\rm{e}}}c={a}^{2}/2$ and transverse momentum is ${P}_{y}/{m}_{{\rm{e}}}c \sim a$. When arriving at the front side of the oblique foil, the laser pulse is reflected. There is an angle of $2\theta$ between the initial and reflected laser pulses. The reflected laser pulse propagates along the −*y* axis for the case of $\theta =45^\circ$. The foil causes the wave break and injection of this thin dense electron layer. At the same time, the laser pulse gets reflected by the foil surface and thus there is no laser-driven wakefield for the injected electron layer. Therefore the electron layer moves forward freely and passes through the foil target. As we consider one electron inside the relativistic electron layer, it interacts with the reflected laser for a distance of about ${\lambda }_{0}/4$ until it reaches the foil front surface. During the interaction, its transverse momentum decreases from ${P}_{y}/{m}_{e}c \sim a$ to ${P}_{y}/{m}_{e}c \sim \,\tan \,\theta$ for the case of $a\gg 1$. Details of the calculation process are shown in the supplementary materials. Therefore, the electrons inside the layer have very homogenous transverse momenta when they reach the foil target. For the simulation case of $\theta =45^\circ$, ${P}_{y}/{m}_{e}c \sim \,\tan (45^\circ )=1$. Figure 2b shows the ${P}_{y}\,$spatial distribution of the electrons inside the layer before and after the laser pulse is reflected. One can see the single electron layer as well as the oblique foil target, which is marked by the dash line. At that time, the upper-part (*y* < 1.7 μm) of laser pulse has already been reflected by the oblique foil while the down-part (*y* < 1.7 μm) is still approaching the foil. At the front surface of the foil, the transverse momentum ${P}_{y}$ of the electron inside the layer drops from ${P}_{y}/{m}_{e}c \sim 15$ to approximately ${P}_{y}/{m}_{e}c \sim 1$, which is in good agreement with the calculated value. It should be also noticed that the electron’s longitudinal momentum increases significantly from ${P}_{x}/{m}_{e}c \sim 29.5$ to ${P}_{x}/{m}_{e}c \sim 40$ after interaction with the reflected laser pulse.

When the dense electron layer finally reaches the foil, these electrons inside the layer have large longitudinal momenta and a transverse momenta of ${P}_{y}/{m}_{e}c\, \sim \,\tan \,\theta$. Therefore it will trigger an intense transverse current^24^. The current is however screened inside the foil target. When it appears at the rear side of the foil, this transverse current of the electron layer emerges again and emits half-cycle pulse radiation^15^. The emitted pulse has only half cycle along the + *y* axis, as same as the electron transverse momentum ${P}_{y}/{m}_{e}c\, \sim \,\tan \,\theta$, and propagates along the *x* axis. Figure 2c plots the electric field ${E}_{y}$ of the emitted attosecond pulse after it propagates in vacuum, 80 μm away from the oblique target. It presents an isolated half-cycle spike with a FWHM width of 285 as. The half-cycle attosecond pulse reaches the maximum electric field ${E}_{y,{\rm{peak}}}=4.2\times {10}^{12}\,{\rm{V}}/{\rm{m}}$, corresponding to the peak intensity of ${I}_{{\rm{peak}}}=n{E}_{y,{\rm{peak}}}^{2}/2{\mu }_{0}c=2.34\times {10}^{18}\,{\rm{W}}/{{\rm{cm}}}^{2}$ with FWHM duration of 200 as. Here $n=1$ and ${\mu }_{0}$ is the magnetic permeability in vacuum. Moreover, the emitted attosecond pulse in our method is automatically phase-stabilized because its amplitude has only a half cycle in + *y* direction, and thus does not require extra phase stabilizing processes in the potential applications^1^.

The generated half-cycle attosecond pulse has a broad spectrum, plotted in Fig. 2d, which mainly covers the visible and part of the ultraviolet range. The radiation spectrum extends up to $35{\omega }_{0}$, limited by the longitudinal resolution and its amplitude has a slow decreasing until the rollover frequency ${\omega }_{{\rm{rs}}}$ of approximately $5{\omega }_{0}$. The high-frequency radiation above ${\omega }_{{\rm{rs}}}$ is not coherent because the electron layer has the longitudinal thickness of a few hundred nanometers. Therefore, the radiation intensity above ${\omega }_{{\rm{rs}}}$ decreases rapidly with increasing frequency. The half-cycle attosecond pulse radiation is mostly in the visible and nearby spectra range, and thus, it is referred to as an optical attosecond pulse. In our method, the peak intensity of the optical attosecond pulse is several orders of magnitude more intense than that generated in the attosecond light-field synthesizer^16^. Such an intense attosecond pulse will make it possible to study the ultrafast electronic quantum dynamics on atomic scale and other attosecond high-field physics.

The radiation intensity of optical attosecond pulse strongly depends on the foil angle $\theta$ as well as peak electron density and the high energy of the dense electron layer ($\sim {n}_{{\rm{e}}}\gamma \,\sin \,\theta$). In the normal incidence case of $\theta =0$, the electrons inside the layer have zero transverse momenta when passing through the oblique foil and thus there is no radiation^25^. The simulation also confirms that there is no visible radiation signal at the case of $\theta =0$, which indicates that the transition radiation of electron layer plays ignorable role in radiation of attosecond pulse. As the foil angle increasing, the transverse momentum of relativistic electrons inside the layer increases and thus the half-cycle pulse intensity increases. The electron layer density plays a critical role in the radiation intensity, where the dense electron layer is resulted from the large longitudinal pondermotive force of the steep laser pulse. Both the initial laser intensity and focal spot size have to be matched to underdense plasma to optimize the electron layer formation. The radiation amplitude of emitted attosecond pulse also relies on the relativistic energy of the electron layer. During momentum modulation by the reflected laser, the electron longitudinal momentum ${P}_{x}/{m}_{e}c$ increases significantly for the *p*-polarized incident laser ca*s*e while it is constant for the *s*-polarized laser case (See electron momentum evolution of electron layer in the supplementary materials). Employing the *p*-polarized incident laser will enhance the radiation intensity. Additionally, the *p*-polarized laser could produce a smaller pulse duration of the attosecond pulse owing to the higher longitudinal momenta of the electrons inside the layer. We also learned from the simulation that the intense half-cycle attosecond pulse is generated with well-structure shape and short pulse duration at the case of foil thickness increasing to 15 μm. Therefore the foil target can be very thick in our proposed mechanism, where the foil is strong enough to handle the large gas pressure in experiments. Moreover, such a thick foil will not allow to transmit the initial laser pulse and the radiation of laser-foil interaction is not able to pass through the thick foil.

**Figure 3:**
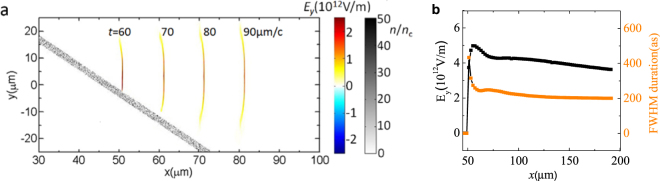
Spatial quality of optical attosecond pulse. (**a**) Spatial distribution of the emitted half-cycle pulse at different time steps, and the oblique foil target. At *t* = 60 μm/*c*, the radiation emission process has not yet finished. (**b**) As the half-cycle attosecond pulse propagates in vacuum, the evolution of its peak electric field and pulse duration are plotted.

The emitted half-cycle pulse gradually separates from the electron layer when they exits the foil as Coulomb expansion of the electron layer will cause a fast decline of the electron density. The half-cycle attosecond maintains its temporal structure in vacuum for a long distance. A remarkable feature of the half-cycle pulse in our method is that the optical half-cycle pulse is highly-collimated with a large transverse size. The generated half-cycle pulse has a transverse size of around 8.6 µm since the electron layer keeps its electron density and narrow thickness in a large transverse range of 10 µm. Figure 3a presents the spatial distribution of the half-cycle pulse at different time steps, the oblique target is also plotted. The half-cycle pulse detaches from the foil target at *t* = 70 μm/*c* with a peak field of 4.4 × 10^12^ V/m and a FWHM transverse size of 8.6 µm. After a propagation distance of 140 µm, the transverse size slowly increases to 10.6 µm, as plotted in Fig. 3b. Additionally, the peak amplitude of the attosecond pulse decreases by only 27% and its pulse duration slightly decreases and then keeps stable at 200 as. It suggests that the half-cycle pulse is highly collimated with a full divergence angle as small as 0.95°, which is crucial for providing intense on-target beam intensities of the attosecond pulse in future applications. The emitted half-cycle pulse carries a total energy of 0.6 mJ, which corresponds to $3.3\times {10}^{-5}\,$of the incident laser energy. Therefore, it reaches a peak power of 2.1 TW. High-intensity attosecond pulse with a small duration of 200 as is suitable for new experiments demonstrating nonlinear time-resolved attosecond dynamical process.

**Figure 4:**
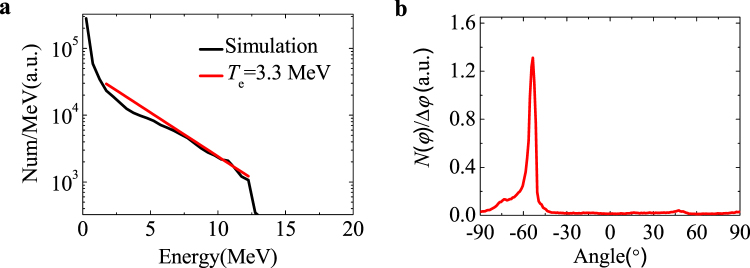
Electrons accelerated by the optical attosecond pulse in the low-density plasma. Energy spectrum (**a**) and angular distribution (**b**) of electrons accelerated by the half-cycle pulse in plasma with a density of ${n}_{{\rm{e}}2}=1\times {10}^{17}{{\rm{cm}}}^{-3}$ at $t=95\mu m/c$.

The strong electric field of the ultra-intense attosecond pulse can immediately ionize light atoms (for example, helium gas) and accelerate electrons to relativistic energies. The second gas cell, as shown in Fig. 1, produces a low-density ${n}_{{\rm{e}}2}$ gas target for ultra-intense half-cycle pulse detection. The dispersion effect of low-density plasma onto the half-cycle pulse is negligible, and thus its temporal structure remains. Since the unipolar half-cycle attosecond pulse is always positive in the time domain (${E}_{y} > 0$), the transverse momenta of excited electrons should be accelerated along the −*y* direction, and therefore the emitted energetic electrons will have a certain angle with the attosecond pulse propagation direction (*x*-axis). The energetic electrons will pass through the thin gas cell wall and be detected, therefore the half-cycle structure of optical attosecond pulse will be verified through the emitted direction measurement of accelerated electrons. We have two gas plasmas of different densities (${n}_{e1}\,$and ${n}_{e2}$) and a tilting plasma foil (${n}_{{\rm{e}},{\rm{f}}}$) in single simulation for attosecond pulse detection, as shown in Fig. 1. The parameters of plasma ${n}_{e1}$ and the foil are taken as same as the case of $\theta =45^\circ$. The low-density plasma is distributed at $60-160{\rm{\mu }}{\rm{m}}$ along the *x*-axis with a homogenous density of ${n}_{{\rm{e}}2}=1\times {10}^{17}{\mathrm{cm}}^{-3}$. In this simulation, the three plasmas are recorded in three different data files so that we can know that which plasma the energetic electrons are coming from. When the half-cycle pulse propagates at *x* = 95 μm, the maximum transverse momentum of accelerated electrons reach ${P}_{y}/{m}_{{\rm{e}}}c \sim -20$, and the longitudinal momentum ${P}_{x}/{m}_{{\rm{e}}}c \sim 15$. Figure 4a displays that the accelerated electrons have a thermal distribution of ${T}_{e}=3.3\,{\rm{MeV}}$ with the cut-off energy of 13 MeV, proving again the extremely high intensity of the attosecond pulse. These high-energy electrons will easily come out of the low-density plasma and be detected. The generation of ultra-intense attosecond pulse makes the study of attosecond relativistic high-field ph*y*sics possible. An important feature of these energetic electrons is that the ${P}_{y}$ distribution is asymmetric. It is restricted to the −*y* domain. The angular distribution of electrons with the energy above 1 MeV in Fig. 4b shows the accelerated electrons are mainly emitted at $\phi =-54.2^\circ$, where $\phi =0^\circ$ is the attosecond pulse propagation direction (*x*-axis). This specific emission angle relies exclusively on the field structure of the half-cycle attosecond pulse, thus hinting at a new method to experimentally verify the proposed mechanism. The emitted angle $\phi$ strongly relies on the peak amplitude of half-cycle attosecond pulse ${E}_{y,{\rm{\max }}}$ and weakly depends on the half-cycle pulse duration. Therefore, the second low-density gas cell with helium gas is present for the detection of the generated ultra-intense optical attosecond pulse. Such high-intensity attosecond pulse provides sufficient photon flux to ionize inner-shell electrons of heavy atoms, offering the ability to observe relativistic inner-shell process. The second gas cell can be also used to study the inner-shell electron dynamics when it is filled with heavy-atom gases.

In summary, a novel method is proposed to generate an isolated ultra-intense half-cycle attosecond pulse with a pulse duration of 200 as based on a relativistic multi-cycle laser pulse irradiating a gas-foil target. The generated attosecond pulse in the visible and near-visible spectral ranges reaches a peak power of 2.1 TW. The method fundamentally releases the strict requirement for the high-contrast laser pulse in the laser-solid interaction^15,26,27^, and the relativistic multi-cycle laser pulse is currently available. Here we have verified the physical scheme by taking one laser-target example through the simulation work and the next step is the experimental demonstration. The gas-foil target in proposed method can be realized based on the specially-designed gas cells and replaceable foils in future experiments. Moreover, a simple way is present to measure the produced half-cycle pulse structure. The high-intensity optical attosecond pulse with a small divergence of 0.95° will provide sufficient photon flux on target to ionize heavy atoms and initiate strong field process. Therefore, this intense optical attosecond pulse generation method offers the ability to investigate nonlinear attosecond optics such as electron correlation as well as ultrafast dynamics inside the atom and molecules^5,28^.

## Electronic supplementary material

Supplementary information
