# Supplementary material for: Terawatt-scale optical half-cycle attosecond pulses
Source: Sci Rep. 2018 Feb 8;8:2669. doi: 10.1038/s41598-018-21052-2 (PMC5805726; doi:10.1038/s41598-018-21052-2)
Supplement: Supplementary file 1 — Supplementary information [file 41598_2018_21052_MOESM1_ESM.pdf]

Supplementary information to "**Terawatt-scale optical half-cycle attosecond pulses**"

Jiancai Xu[1], Baifei Shen[1, 2, 3], Xiaomei Zhang[1], Yin Shi[1], Liangliang Ji[1], Lingang Zhang[1], Tongjun Xu[1], Wenpeng Wang[1], Xueyan Zhao[1] and Zhizhan Xu[1]

[1]State Key Laboratory of High Field Laser Physics, Shanghai Institute of Optics and Fine Mechanics, Chinese Academy of Sciences, P. O. Box 800-211, Shanghai 201800, China
[2]Department of Physics, Shanghai Normal University, Shanghai 200234, China
[3]Collaborative Innovation Center of IFSA (CICIFSA), shanghai Jiao Tong University, Shanghai 200240, China

1. Single thin dense electron layer formation

The intense femtosecond laser pulse drives multi-periodic plasma waves when it propagates into the underdense plasma. Fig M-1(a-f) plots the laser field, laser-driven wakefield as well as the electron density distribution of the plasma waves. At the same time of driving the plasma waves, the longitudinal pondermotive force of intense laser pulse pushes background electrons forward along the propagation direction to relativistic energies. Multiple electron layers form in the front part of the first plasma period, and co-move with the laser pulse, as shown in Fig. M-1(c). When the laser pulse further propagates, the laser front steeps itself because of self-etching effect. At $t = 167$ fs, the laser pulse loses its front and transforms to a step-like pulse, as plotted in Fig. M-1(d). Therefore, the longitudinal pondermotive force of intense laser pulse increases greatly. There is a thin dense layer of relativistic electrons co-moving with the laser front (marked in Fig. M-1(f)). The electron density spike has a peak value of $0.7n_c$ with a narrow full-width-half-maximum (FWHM) thickness of 320 nm. Moreover, this dense electron layer has good transverse uniformity over a length of more than 10 μm because of the large laser focal spot size. These electrons inside the dense electron layer are directly accelerated by the intense laser pulse, therefore their momenta approximately follow the local vector potential $a(x, t)$, $P_x/m_ec \sim a^2/2$ and $P_y/m_ec \sim a$, as shown in Fig. M-1(g, h).

2. Half-cycle pulse radiation

When an electron layer has a velocity perpendicular to itself, there are magnetic and electric fields emitting out from the electron layer[1]. In our case, an intense electron layer passes through the foil and triggers a transverse current due to certain transverse momentum of the electrons inside the layer. The radiation emits at the rear side of foil target since the current is screened inside the foil target. Here we assume that the electron momentum does not significantly change inside the foil since the foil thickness is only a few micrometers. We calculate the momentum of electrons inside the dense layer when it reaches the foil.

The motions of electrons follow single electron model in the laser field[2]. For the p-polarized case, $a_y = a_0\sin(\pi t/\tau_0)$, and $a_z = 0$. Therefore the electrons have $P_{x0}/m_ec = a_0{}^2/2$, $P_{y0}/m_ec = -a_0$, and $\gamma = 1 + a_0{}^2/2$ when they get accelerated in the incident laser field. After the laser pulse gets reflected by the flat foil, the laser pulse propagates in x'-axis with the polarization direction in the y'-axis, as shown in Fig. M-2. The electrons inside the layer will

interact with the reflected laser front until they reach the foil. We will calculate the electron momentum modulation by the reflected laser.

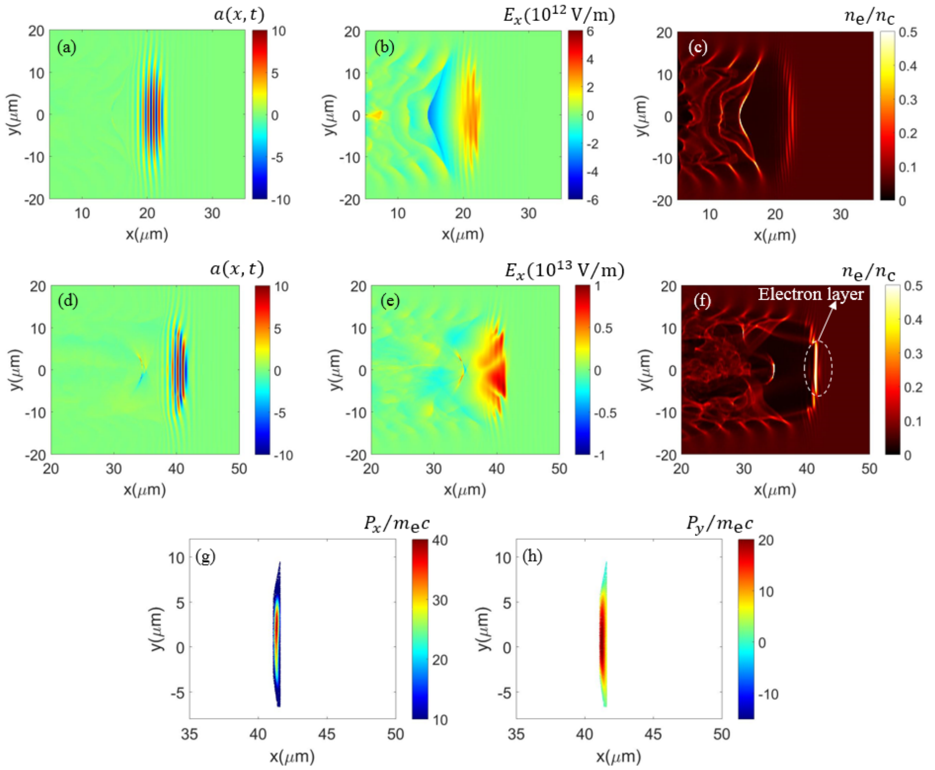

Figure M-1| (a-f) evolution of laser electric field (local vector potential, $a(x,t)$), longitudinal electric field (E_x) and electron density (n_e) with different time steps of $t = 100$ fs and $t = 167$ fs. (g, h) the longitudinal and transversal momenta distribution of the electrons inside the thin layer at $t = 167$ fs.

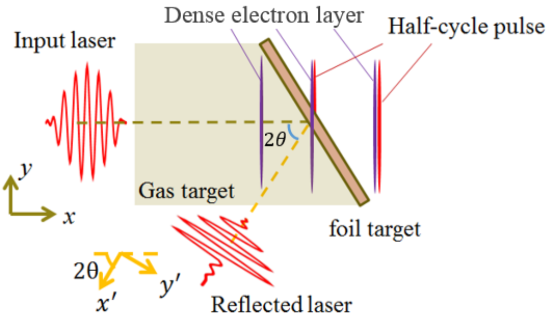

Figure M-2| **Physical scheme of the half-cycle attosecond pulse emission**

The *x*-*y* frame shifts to $x' - y'$ frame. Before the electrons get interacted with the reflected laser, they have initial momenta in x'-axis and y'-axis

$$P_{x'0} = -P_{y0}\sin(2\theta) - P_{x0}\cos(2\theta)\,, P_{y'0} = -P_{y0}\cos(2\theta) + P_{x0}\cos(2\theta) \qquad (1)$$

Therefore the electrons have κ=γ $- P_{x'0}$, and in the reflected laser field, the electrons have

$$P_{x'} = \frac{1+P_{y'}^2}{2\kappa} - \frac{\kappa}{2}\ ,\ P_{y'} = P_{y'0} + a_1. \qquad (2)$$

As assuming the reflected laser has the same amplitude of the incident laser $a_1 = a_0$. We can calculate the final electron momenta after interaction with of the reflected laser pulse based on eq.

(1) and (2) and transfer back to x-y frame

$$P_{x,p} = P_{y'}\sin(2\theta) - P_{x'}\cos(2\theta) = \frac{a_0}{2}\frac{a_0(6+a_0^2)+a_0(a_0^2-2)\cos(2\theta)+4(1+a_0^2)sin(2\theta)}{2+a_0^2+a_0^2\cos(2\theta)+2a_0\sin(2\theta)}. \quad (3)$$

$$P_{y,p} = -P_{x'}\sin(2\theta) - P_{y'}\cos(2\theta) = \frac{2a_0\sin\theta(a_0\cos\theta+2\sin\theta)}{2+a_0^2+a_0^2\cos(2\theta)+2a_0\sin(2\theta)} \quad (4)$$

For the case of $a_0 \gg 1$, The transverse momenta can be simplified to $P_{y,p}/m_e c \sim \tan\theta$, which indicates that the transverse momentum of the electron only depends on the foil oblique angle when it reaches the foil.

By using the same method, we can calculate the final momenta of electrons for s-polarized incident laser pulse ($a_y = 0$, and $a_z = a_0 \sin(\pi t/\tau_0)$). The electrons have the momenta in incident laser field $P_{x0}/m_e c = a_0^2/2$, $P_{y0}/m_e c = 0$ and $P_{z0}/m_e c = a_0$. After the interaction with the reflected laser pulse, the electron momentum are

$$P_{x,s} = \frac{a_0^2(2+a_0^2)\cos^2(\theta)}{2+a_0^2+a_0^2cos(2\theta)} \quad (5)$$

$$P_{y,s} = \frac{2a_0^2\sin(2\theta)}{2+a_0^2+a_0^2cos(2\theta)} \quad (6)$$

The transverse momentum of electron is simplified to $P_{y,s}/m_e c \sim \tan\theta$ as $a_0 \gg 1$. Therefore the transverse momentum of electron inside the layer $P_{y,s}/m_e c \approx P_{y,p}/m_e c \sim \tan\theta$ depends on the foil angle. The calculation value for P_y of the electron inside the layer has a good agreement with the simulation data, as shown in Fig. M-3(a). The sudden large change of transverse momentum of electron during the interaction of the reflected laser pulse before the foil will trigger synchrotron radiation in keV scale[3], but the radiation is not coherent in our case because the longitudinal width of electron layer is a few hundred nanometer, which is too large for the radiation wavelength 4. Therefore the keV-scale x-ray radiation is negligible for the generated half-cycle pulse at the rear side of the foil target. Meanwhile, the electron longitudinal momentum P_x has also modulated by the reflected laser. Fig. M-3(b) show that P_x increases significantly for the p-polarization case of incident laser pulse while it keeps constant for the s-polarization case.

When the electron layer reaches the foil, the electrons have large longitudinal momenta and a transverse momenta of $P_y/m_e c \sim \tan\theta$. It will trigger a transverse current since it has a transverse velocity perpendicular to itself. The transverse current however is screened inside the foil. When it emerges again at the rear side of the foil, it will emit a half-cycle pulse[5]. The electron transverse momentum decreases due to the radiation dumping. The half-cycle pulse intensity ($\sim n_e \gamma \sin\theta$) increases with the incident angle as well as the electron energy. For the fixed the laser pulse and gas target, Series of simulations have been performed to scan the foil oblique angle for different polarization of the incident laser. The corresponding results, as plotted in Fig. M-3(c), show that the radiation intensity increases with the oblique angle and the employed p-polarized laser pulse will enhance the radiation intensity compared to s-polarized laser. Fig. M-3(d) presents that the pulse duration of half-cycle attosecond pulse decreases slightly with the foil oblique angle for p-polarization case but it increases with the foil oblique angle for s-polarization case. Moreover, the half-cycle based on p-polarized laser pulse has much shorter pulse duration than the s-polarized case. The electron layer gets accelerated by the reflected laser pulse longitudinally and they have higher longitudinal momenta P_x and thus the emitted half-cycle pulse has shorter duration ($\tau \sim 1/\gamma$).

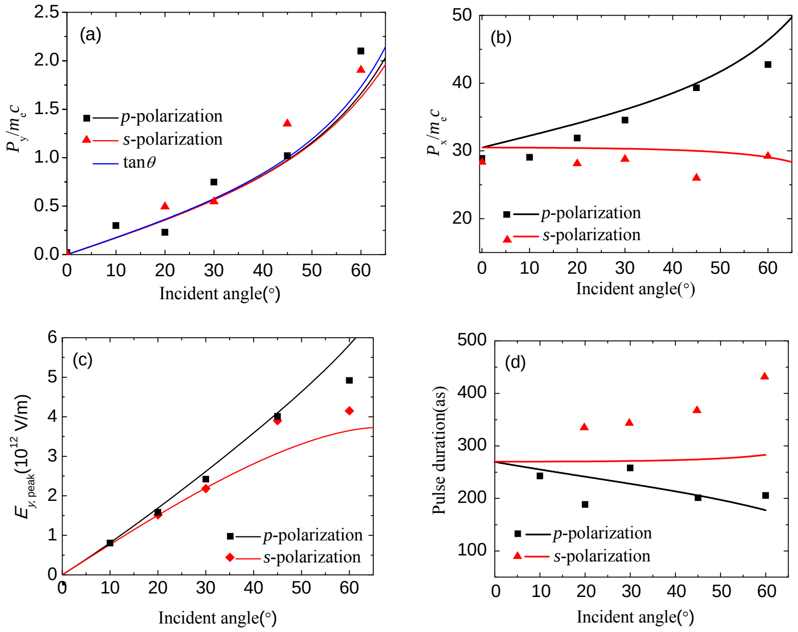

Figure M-3│ comparisons of analytical model and simulation result: transverse (**a**) and longitudinal (**b**) momentum of relativistic electrons inside the layer after interaction with the reflected laser pulse. The peak electric field value (**c**) and FWHM pulse duration (**d**) of the emitted half-cycle attosecond pulse depends on the foil oblique angle

3. Half-cycle pulse detection in low-density plasma

2D simulations indicate that the low-density plasma has ignorable dispersion effect on the half-cycle pulse when the electron density is lower than $5 \times 10^{17}\text{cm}^{-3}$. Therefore the half-cycle attosecond pulse keeps its temporal structure in underdense plasma with the density of $1 \times 10^{17}\text{cm}^{-3}$, as plotted in Fig. M-4 (a, b).

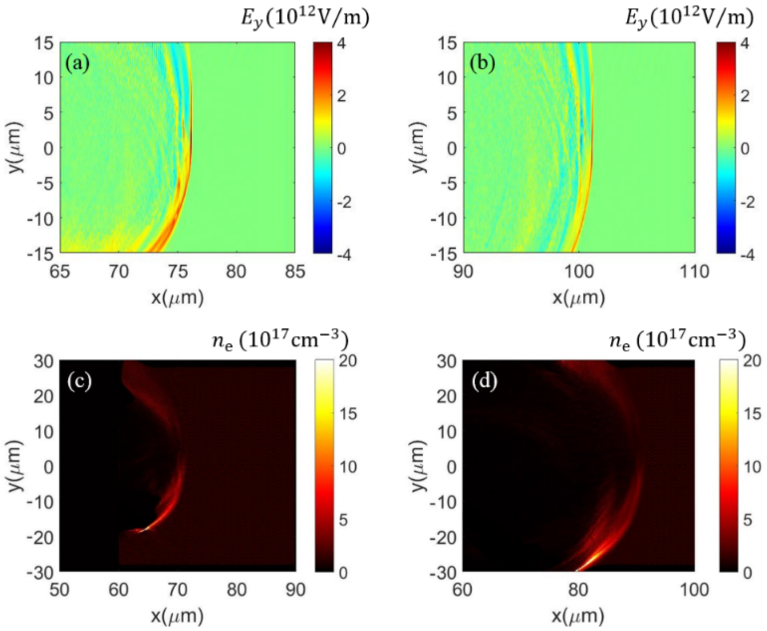

Figure M-4 │ The half-cycle pulse propagation in low-density plasma and it accelerates background electrons. (a, b) Electric field (E_y) of radiated half-cycle pulse inside the low-density

plasma with a density of $n_{\mathrm{e}2} = 1 \times 10^{17}\mathrm{cm}^{-3}$ at different time steps of $t = 283\mathrm{fs}$ and $t = 367\mathrm{fs}$. (c, d) electrons density accelerated by half-cycle attosecond pulse at $t = 283\mathrm{fs}$ and $t = 333\mathrm{fs}$.

Background electrons are directly accelerated by the intense attosecond pulse, therefore most of electrons move to –y direction because of half-cycle pulse ($E_y > 0$ for the whole pulse). Fig. M-4(c, d) confirms that the electron density has obvious asymmetry distribution along y axis, where the electron density peak moves to –y direction. The angular distribution of energetic electrons with the energy above 1 MeV shows the accelerated electrons are mainly emitted at $\varphi = -54.2°$, where $\varphi = 0°$ is the attosecond pulse propagation direction (x axis). This specific emission angle of fast electrons relies exclusively on the field structure of the half-cycle attosecond pulse, thus bringing a new method to experimentally verify the optical attosecond pulse. Moreover, the spectrum of these electrons has a thermal distribution of $T_{\mathrm{e}} = 3.3\ \mathrm{MeV}$ with the cut-off energy of 13 MeV. These high-energy electrons will easily come out of the low-density plasma and be detected.

The emitted angle φ strongly relies on the peak amplitude of half-cycle attosecond pulse $E_{y,\max}$ and weakly depends on the half-cycle pulse duration. We have also numerically calculated the signal electron acceleration process by the half-cycle attosecond pulse by using Mathematica, which allows us to scan the dependence of emitted angle φ on peak amplitude $E_{y,\max}$ as well as the pulse duration of the half-cycle attosecond pulse. As plotted in Fig. M-5, the emitted angle φ drops rapidly with increasing pulse amplitude $E_{y,\max}$ of half-cycle pulse in a large range between -80° and -30°. The curves between φ and $E_{y,\max}$ with different pulse durations show similar changes in trend and the values of φ increases with the pulse duration. These results again confirm that is it is able to identify the half-cycle attosecond pulse generation through the specific emission angle of accelerated electrons.

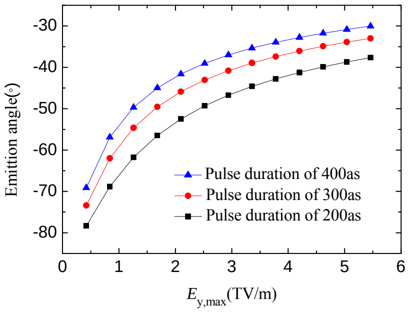

Figure M-5 | Emission angle φ of accelerated electrons has strong relation with pulse amplitude as well as pulse duration of half-cycle attosecond pulse

We also learned from the simulation that the original electron bunch ($n_{\mathrm{e}1}$) has ignorable effect on the $n_{\mathrm{e}2}$ electron detection and thus half-cycle pulse detection. After the original electron bunch passes through the foil, its density has a fast drop because of Coulomb expansion of the electron layer. While the half-cycle pulse keeps its amplitude and structure for a long distance. Therefore the half-cycle pulse will accelerate background electrons in low-density $n_{\mathrm{e}2}$ region and the original electron bunch has ignorable contribution on the electron acceleration. Figure M-6(a) plots the spatial distribution of the original electron bunch at $t = 333\mathrm{fs}$ and confirms that it has comparable density with the background plasma, which has no significant effect on the electron acceleration in $n_{\mathrm{e}2}$ plasma. Moreover, we also plotted the angular distribution of original electron bunch, as shown in Fig. M-6(c), which occupies different angle with low electron number from the accelerated

electrons in n_{e2} plasma. Therefore the original electron bunch (n_{e1}) has ignorable influence on the n_{e2} electron detection and thus half-cycle pulse detection.

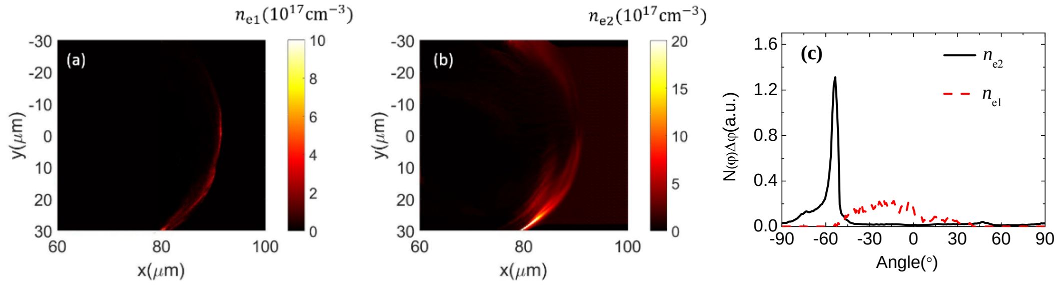

Figure M-6 | the original electron bunch (n_{e1}) has ignorable influence on the half-cycle pulse detection. (a) spatial distribution of original electron bunch and (b) electrons density accelerated by half-cycle attosecond pulse at $t = 333$fs.(c) Angular distribution of original electron bunch(n_{e1}) and accelerated electrons in n_{e2} plasma region.

Reference

1. Feynman, R. P., Leighton, R. P. & Sands, M. The Feynman lectures on Physics, Vol. 2(Addison-Wesley, Reading, MA, 1966) Section 18-4.
2. Meyer-ter-Vehn, J. & Wu, H. C. Coherent Thomson backscattering from laser-driven relativistic ultra-thin electron layers. *Eur. Phys. J.* D 55, 433-441 (2009).
3. Phuoc, K. Ta. *et al*. All-optical Compton gamma-ray source. *Nat. Photon*. 6, 308-311(2012).
4. Jackson, J. D. Classical Electrodynamics (John Wiley&Sons, 1999).
5. Wu, H. C. & Meryer-ter-Vehn, J. Giant half-cycle attosecond pulses. *Nat. Photon*. 6,304-307 (2012).
